# Cardiovascular risk estimated after 13 years of follow-up in a low-incidence Mediterranean region with high-prevalence of cardiovascular risk factors

**DOI:** 10.1186/1471-2458-10-640

**Published:** 2010-10-25

**Authors:** José M Huerta, María-José Tormo, Diana Gavrila, Carmen Navarro

**Affiliations:** 1 Department of Epidemiology, Murcia Regional Health Authority, Ronda de Levante 11, 30008 Murcia, Spain; 2 CIBER Epidemiología y Salud Pública (CIBERESP), Spain; 3 Department of Sociosanitary Sciences, University of Murcia School of Medicine, Spain

## Abstract

**Background:**

Murcia (south-east Spain) shows increased cardiovascular (CV) morbimortality as compared to other Spanish regions. Our objective was to assess the CV risk associated with major risk factors (RF) among adult population of Murcia.

**Methods:**

A cohort of 2314 subjects (18-70 years) with full biochemical and questionnaire data was followed-up for 13 years. Incident cases of ischemic heart disease and stroke were identified by record linkage, individual questionnaires and revision of medical records. Relative risks were obtained by multivariate Cox regression stratified by age and sex, and ischemic risk attributable to CVRF was calculated.

**Results:**

After more than 26276 person-years of follow-up, 57 incident ischemic events (77% men) and 37 stroke cases (62% men) were identified. Independent risk factors of ischemic heart disease (IHD) and all CV events combined, with RR ranging from 1.6 to 2.6, were total serum cholesterol ≥ 240 mg/dl (HR = 2.6, 95%CI:1.3-5.1), blood pressure levels ≥ 140/90 mmHg (HR = 2.6, 95%CI:1.4-4.8), ever tobacco smoking (HR = 2.2; 95%CI:1.1-4.5), and diabetes (HR = 2.0; 95%CI: 1.0-3.8). No increased CV risk was detected for known participants under treatment who showed cholesterol and blood pressure values below the clinical risk threshold. Smoking was significantly associated with stroke. For all events combined, the major risk factors were hypercholesterolemia, hypertension and ever use of tobacco. Despite its high prevalence, obesity was not associated to CV risk. Most of the IHD cases were attributable to smoking (44%), hypertension (38%) and hypercholesterolemia (26%).

**Conclusions:**

In the Region of Murcia, smoking accounted for the largest proportion of cardiovascular risk, whereas hypertension displaced hypercholesterolemia as the second leading cause of CV disease. Our study deepens in our understanding of the cardiovascular epidemiology in Spanish areas of Mediterranean Europe with relatively high cardiovascular morbimortality, that are poorly represented by the available risk equations.

## Background

The Spanish Region of Murcia is located in the Mediterranean basin, one of the areas with the lowest rates of cardiovascular disease (CVD) mortality in Europe [1]. However, the burden of CVD is high in Murcia as compared to other Spanish regions [2], yet still low in an international context. The prevalence of CVRF is also high, as characteristic in Spain [3-5], albeit the diet preserves cardiovascular-healthy Mediterranean traits [6]. Cardiovascular prevention is a Public Health priority in the region, as well as the rest of the country.

The evaluation and prioritization of Public Health interventions for controlling CVD rely on estimates of *population risk *attributable to CVRF [7]. Obtaining such indicators requires knowing in anticipation both the prevalence of RF in the community and the associated relative risks, ideally coming from population-based prospective studies. From an *individual *perspective, however, charts of coronary risk are used to predict the chance of suffering a CV event within a certain period of time [8-10]. Those nowadays available in Spain, however, rely on risk estimates available for the USA (REGICOR) [8] or European (SCORE) [9] populations. Both, REGICOR and SCORE are calibrations of the Framingham and SCORE risk functions, respectively, but only the first has been validated for use in Spain [11]. However, a specific risk chart for the country is not yet available that takes regional variability into account, in spite of the reiterated claims that risks systematically used in clinical practice [8-10] overestimate the CV risk of the Spanish population [11,12].

The scarce prospective literature available in Spain [13,14] confirms an elevated IHD risk for hypercholesterolemia and tobacco smoking, whereas associations for hypertension, overweight or diabetes are less consistent.

The objective of the present study was to estimate specific CVD risk associated with major CVRF and the attributable risk of IHD in a representative sample of population from Murcia [15].

## Methods

### Study sample and design

**Figure 1:**
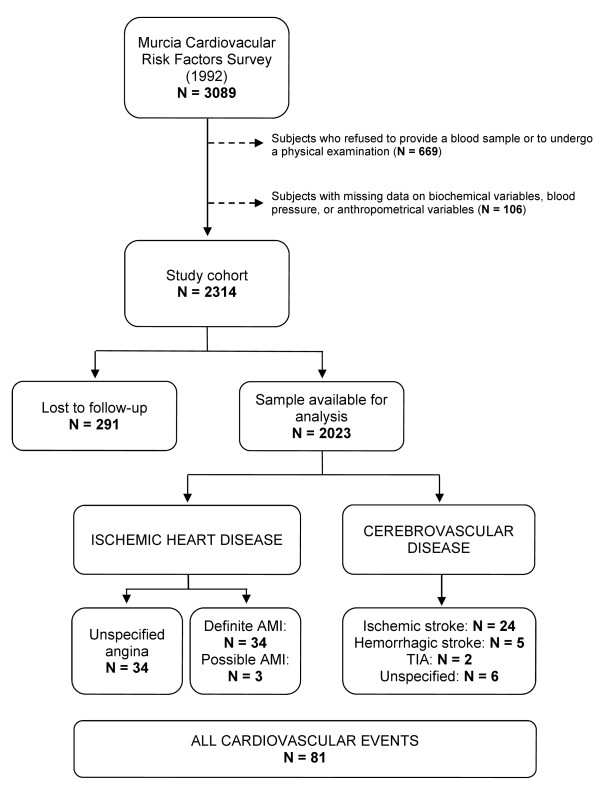
**Study sample flow-chart**. Definite AMI criterion based on the MONICA diagnostic classification [18]. AMI: acute myocardial infarction. TIA: transient ischemic attack.

This longitudinal prospective population-based study comprises a cohort of 3089 men and women, aged 18-70 years, who were recruited from November 1991 until March 1993. A random representative sample of the Murcia population was selected with clustering by (administrative) health area and sampling quota were defined by type of residence (rural/urban), age group and sex, with the aim of surveying the regional prevalence of CVRF. Participants were contacted by mail, telephone or home visit. Baseline questionnaire information was obtained on clinical and lifestyle characteristics and subjects were invited to undergo a physical examination. Furthermore, 78% of participants provided a fasting blood sample for serum lipid analysis (Figure 1). A more detailed description of the methodology has been published elsewhere [15].

All participants voluntarily agreed to take part in the study, in compliance with the Declaration of Helsinki. The information collected was treated as confidential and the databases were registered at the Spanish Data Protection Authority as stipulated by law. The study was conducted with the approval of the Ethics Committee of the Virgen de la Arrixaca Hospital (Murcia), the main regional hospital.

### Baseline data collection

A questionnaire administered by trained personnel was used to gather information on sociodemographic, lifestyle and health-related factors. Interviews took place in primary care institutions and local public centers. Questions on medical history and smoking habit included were those of the MONICA study [16]. The highest academic attainment of participants (illiterate, non-primary, primary, secondary, or higher) was registered. Recent physical activity for the last two weeks was assessed by asking the frequency and duration of moderate and vigorous sporting activities, by separate, using a validated questionnaire [17]. During a physical examination, participants had their blood pressure measured and height and weight registered according to MONICA standards. Calibrated mercury sphygmomanometers (Riester 660/306, model Diplomat, Germany) were used for blood pressure measurement and systolic and diastolic values were defined as the mean of two consecutive readings. Weight and height were assessed for subjects barefoot in light clothing, using calibrated equipment. Body mass index (BMI) was calculated as weight (in kg) divided by square height (in m).

Total serum cholesterol and triglyceride concentrations were analysed by enzymatic methods in a Hitachi autoanalyzer (model 717) at the Department of Clinical Biochemistry of the Virgen de la Arrixaca University Hospital (Murcia). The apparatus coefficients of variation were 3.0-3.8% for total cholesterol and 2.1-2.2% for triglycerides.

### Definition of risk factors

Hypercholesterolemia was defined as total serum cholesterol ≥240 mg/dl (6.2 mmol/l), a previous diagnosis of elevated cholesterol or prescribed use of lipid-lowering drugs. Hypertriglyceridemia was defined as serum triglycerides ≥150 mg/dl (1.7 mmol/l). Hypertension was defined as mean systolic blood pressure ≥140 mmHg or mean diastolic blood pressure ≥90 mmHg, a previous diagnosis of hypertension or use of anti-hypertensive medication. 'Ever smokers' included current and former smokers. Diabetes and use of anti-diabetic drugs were self-reported. Normal weight (<25 kg/m^2^), overweight (25-29.9 kg/m^2^) and obesity (≥30 kg/m^2^) were defined according to BMI.

### Follow-up and identification of CVD cases

Retrospective information on newly diagnosed cases of angina, AMI and stroke, and vital status of the cohort, was collected between November 2005 and June 2006 for more than 99% of the sample, completing a mean follow-up of 13.1 (± 1.5) years. Occurrence and dates of new cardiovascular events were recorded for 2023 participants after excluding subjects with missing data on study variables (N = 775) and those lost to follow-up (N = 291; 12.6%).

The identification of cases was carried out through a multiple strategy: a) contact with participants by postal or telephone interview to register self-reported risk factors or CV events diagnosed by a physician, and dates and causes of hospital admissions during the last ten years; b) record linkage with hospital discharge databases of all public hospitals in Murcia and the regional Mortality Register; c) revision of the medical history of all participants with hospital admission due to any of the following ICD-9 codes: 250-250.93, 272-272.9, 278.0-278.01, 401-405.99, 410-414.9, 420-429.9, 430-438.9, or 797-799.9; d) revision of the medical history, either in the corresponding primary care centre or the reference hospital, of all dead participants due to any of the following ICD-10 codes: E10-E11.9, E66-E66.9, E78-E78.9, G45-G46.8, I10-I11.9, I20-I25.9, I46-I46.9, I50-I50.9, I61-I64, I67-I67.9, R96-R96.1, or R98, as well as all those participants alive who self-reported any risk factor or CV event in the follow-up interview. Events were classified according to MONICA criteria [18].

Cases were censored at the date of diagnosis of the CV event. Non-cases were censored at date of a non-cardiovascular death or at follow-up data collection.

### Statistical analyses

The distribution of variables by sex was summarised as median values and interquartile range or percentages. Because of the lack of normality of continuous variables, the Kruskall-Wallis test was used to check for statistical differences in the distribution of data between men and women, whereas χ^2 ^test were used to assess whether categorical variables were proportionally distributed by sex.

Hazard ratios (HR) of CVD by levels of risk factors were obtained using Cox regression models with age as the underlying time variable. The proportional-hazards assumption was tested on the basis of Schoenfeld residuals using the *estat phtest *command in STATA. No significant violations of the proportionality assumption were detected. Due to the limited number of cases among women, sex was used as a stratifying variable in Cox models instead of presenting disaggregated results for men and women. Regression models were further stratified by 5-year age categories in order to account for differences in baseline hazard functions by age group. Trend *P *values for cholesterol and BMI levels were computed by entering the variables in the models as continuous with increasing categories coded by consecutive integer values. Heterogeneity by sex in CVD risk for each risk factor was established on the basis of the *P *value of a likelihood ratio test between models with and without the *risk factor × sex *interaction term.

Prevalent cases of IHD (N = 23) were excluded from the analysis of angina and AMI end-points, whereas participants with a prevalent stroke (N = 8) were excluded from models of cerebro-vascular risk. The analysis of all CV events combined finally excluded all participants with any prevalent CV condition at baseline and censored their follow-up time at the date of diagnosis of the first CV event.

The Population Attributable Risk (PAR) was calculated according to the formula: *p*_*E*_(RR - 1)/(1 + *p*_*E*_(RR - 1)) [19], where *p*_*E *_denotes the prevalence of exposition to every individual risk factor based on results of the DINO Study (2002) [5], the most updated population-based survey on chronic morbidity available in Murcia, and RR denotes the multivariate-adjusted relative risk as estimated by the HR of multivariate Cox regression in the present study [15]. In order to prevent PAR estimates from being influenced by age, risk factor prevalences were standardised to the age distribution of the Murcia population according to the 2001 census and truncated to the age range of the current study (20-70 years).

All analyses were performed with STATA/SE version 10.1. Level of statistical significance was set at 5%.

## Results

A total of 71 incident cases of IHD were identified (34 cases of angina and 37 cases of AMI). Fourteen AMI cases (38%) had had a previous episode of angina. Furthermore, a cerebro-vascular event was diagnosed in 37 participants (Figure 1). Three out of four ischemic events (77%) and two out of three stroke cases detected (62%) were in men

Table 1 summarises relevant clinical variables by sex. On average, participants were middle-aged (≈40 years) and overweight. The distribution of CV risk factors differed by sex, except for self-reported diabetes. Men showed a less favourable lipid profile than women, higher prevalence of hypertension, and more frequently engaged in sporting activities. Among those people that reported being under treatment either for high serum cholesterol or blood pressure, men were more often found uncontrolled. A lower percentage of women had completed secondary or university studies, and fewer women smoked. Although no overall differences in average body mass index were observed by sex, a higher percentage of men were overweight whereas a larger proportion of women were obese.

**Table 1:** Distribution of cardiovascular risk factors by sex among cohort participants (≥18 years)

		**Men (N = 956)**		**Women (N = 1067)**		
	
		**Median**	**IQR**	**Median**	**IQR**	***P***
	
**Age at recruitment (years)**		39.3	19.5	40.5	19.0	0,699
**Body mass index (kg/m**^**2**^**)**		26.7	4.7	26.3	6.6	0,572
**Total serum cholesterol (mg/dl)**		196.0	63.5	186.0	63.0	<0,001
**Serum triglycerides (mg/dl)**		118.0	86.5	84.0	51.0	<0,001
**Systolic blood pressure (mmHg)**		129.0	20.0	121.0	24.0	<0,001
**Diastolic blood pressure (mmHg)**		79.0	16.0	76.0	16.0	<0,001
		**N**	**%**	**N**	**%**	***P***
	
**Hypercholesterolemia**^**a**^	No	664	69.5	792	74.2	
	Controlled	100	10.5	105	9.8	
	Uncontrolled	164	17.2	143	13.4	0.040
	Unknown^d^	28	2.9	27	2.5	
	
**Triglyceridemia**	<150 mg/dl	650	68.0	943	88.4	
	≥150 mg/dl	306	32.0	124	11.6	<0,001
	
**Educational level**	Illiterate	19	2.0	47	4.4	
	Incomplete primary	255	26.7	374	35.1	
	Primary shool	394	41.2	428	40.1	
	Secondary school	165	17.3	130	12.2	
	University	123	12.9	88	8.2	<0,001
	
**Hypertension**^**b**^	No	562	58.8	695	65.0	
	Controlled	60	6.3	101	9.4	
	Uncontrolled	310	32.4	265	24.8	<0,001
	Unknown^d^	24	2.5	9	0.8	
	
**Diabetes**^**c**^	No	859	89.9	945	88.6	
	Yes	74	7.7	103	9.7	0,140
	Unknown^d^	23	2.4	19	1.8	
	
**Smoking**	Non-smoker	309	32.3	740	69.4	
	Former smoker	132	13.8	37	3.5	
	Smoker	515	53.9	290	27.2	<0,001
	
**Body mass index**	<25 kg/m^2^	293	30.7	413	38.7	
	25-29.9 kg/m^2^	485	50.7	378	35.4	
	≥30 kg/m^2^	178	18.6	276	25.9	<0,001
	
**Moderate physical activity**	None	722	80,8	972	91,1	
	<1/2 h/day	97	10,2	54	5,1	
	≥1/2 h/day	87	8,1	41	3,8	<0,001
	
**Intense physical activity**	None	830	86,8	1011	94,8	
	<2 h/day	40	4,2	19	1,8	
	≥2 h/day	86	9,0	37	3,5	<0,001

IQR: Interquartile range (Q_3 _- Q_1_).

^a^Controlled hypercholesterolemia defined as prior diagnosis of hypercholesterolemia or use of lipid-lowering medication and total serum cholesterol <240 mg/dl; uncontrolled hypercholesterolemia defined as total serum cholesterol ≥240 mg/dl.

^b^Controlled hypertension defined as prior diagnosis of hypertension or use of anti-hypertensive medication and systolic/diastolic blood pressure <140/90 mmHg; uncontrolled hypertension defined as systolic/diastolic blood pressure ≥140/90 mmHg.

^c^Defined as a self-reported diagnosis of diabetes or prescription of insulin or oral anti-diabetic medication.

^d^Participants with missing data on drug use, excluded from statistical tests.

Table 2 shows the hazard ratios of cardiovascular events by defined categories of CVRF. Overall, IHD was associated to elevated serum cholesterol and blood pressure levels, diabetes and present or past smoker condition. In separate analyses, higher lipid concentrations were significantly associated with AMI, whereas no factor reached statistical significance as predictor of angina. Smoking was additionally strongly associated to stroke. For all CV events combined, smoking and hypertension were independently and significantly associated with an elevated risk of suffering a CV event of any kind. A possible association with hypertriglyceridemia and diabetes remained above the threshold of, but close to, statistical significance. Overweight or obese participants showed no increased CV risk in this cohort neither in univariate nor in fully adjusted models. Body mass index was also tested as a continuous or binary (non-obese/obese) variable and results did not substantially change. As for gender differences in CV risk profile, heterogeneity by sex was lower for stroke than it was for IHD.

**Table 2:** Risk of incident cardiovascular disease in a cohort of adult population from Murcia (south-east Spain)

	Ischemic heart disease						Stroke		All events	
					
	Angina		AMI		All ischemic events					
					
	Py/cases	HR (95% CI)	Py/cases	HR (95% CI)	Py/cases	HR (95% CI)	Py/cases	HR (95% CI)	Py/cases	HR (95% CI)
	
Total cholesterol (mg/dl)										
<200	15393/10	1	15404/8	1	15381/16	1	15454/19	1	15308/31	1
200-239	7154/8	1.14 (0.43-3.05)	7182/7	0.83 (0.29-2.39)	7138/12	0.97 (0.44-2.11)	7232/9	0.54 (0.23-1.28)	7104/18	0.71 (0.39-1.29)
≥240	3814/16	2.27 (0.91-5.68)	3813/22	2.85 (1.16-6.99)	3757/29	2.59 (1.30-5.14)	3913/9	0.79 (0.33-1.90)	7105/32	1.61 (0.92-2.81)
*P *for trend (categorical)		*P *= 0.075		*P *= 0.009		*P *= 0.005		*P *= 0.459		*P *= 0.119
					*P*_heterogneity by sex _= 0.071		*P*_heterogneity by sex _= 0.500		*P*_heterogneity by sex _= 0.024	
**Triglycerides (mg/dl)**										
<150	20902/17	1	20947/14	1	20868/28	1	21041/26	1	20768/44	1
≥150	5459/17	1.64 (0.77-3.50)	5452/23	2.48 (1.17-5.27)	5408/29	1.64 (0.91-2.94)	5558/11	0.83 (0.39-1.79)	5369/37	1.52 (0.94-2.47)
					*P*_heterogneity by sex _= 0.035		*P*_heterogneity by sex _= 0.003		*P*_heterogneity by sex _= 0.009	
										
**Blood pressure (mm Hg)**										
<140/90	19212/12	1	19151/9	1	19103/17	1	19258/13	1	19067/25	1
≥140/90		2.12 (0.95-4.71)	7248/28	3.68 (1.62-8.34)	7172/40	2.59 (1.39-4.84)	7341/24	1.92 (0.91-4.01)	7070/56	2.44 (1.46-4.07)
					*P*_heterogneity by sex _= 0.040		*P*_heterogneity by sex _= 0.964		*P*_heterogneity by sex _= 0.101	

**Diabetes**^**a**^										
No	23620/23	1	23627/28	1	23553/40	1	23820/29	1	23449/61	1
Yes	2179/9	2.04 (0.95-4.71)	2199/9	1.77 (0.79-3.97)	2162/15	1.98 (1.02-3.83)	2216/7	1.45 (0.60-3.47)	2135/18	1.67 (0.96-2.92)
					*P*_heterogneity by sex _= 0.004		*P*_heterogneity by sex _= 0.483		*P*_heterogneity by sex _= 0.010	

**Ever smoking**										
No	13623/15	1	13660/13	1	13597/21	1	13723/16	1	13526/32	1
Yes	2070/19	1.93 (0.78-4.74)	12739/24	1.94 (0.84-4.51)	12679/36	2.22 (1.10-4.46)	12875/21	3.88 (1.31-11.50)	12610/49	2.39 (1.31-4.38)
					*P*_heterogneity by sex _= 0.010		*P*_heterogneity by sex _= 0.716		*P*_heterogneity by sex _= 0.030	

**BMI categories**^**b**^										
Normal weight	9328/4	1	9324/5	1	9317/7	1	9387/4	1	9306/10	1
Overweight	11221/18	1.07 (0.33-3.41)	11232/24	0.82 (0.29-2.33)	1176/33	1.06 (0.44-2.52)	11346/16	2.61 (0.72-9.42)	11114/43	1.29 (0.62-2.66)
Obese	5811/12	0.91 (0.26-3.18)	5843/8	0.38 (0.11-1.27)	5783/17	0.76 (0.29-1.99)	5866/17	2.56 (0.69-9.48)	5717/28	1.08 (0.50-2.34)
*P *for trend (categorical)		*P *= 0.801		*P *= 0.065		*P *= 0.455		*P *= 0.216		*P *= 0.950
					-^c^		-^c^		-^c^	

AMI: Acute myocardial infarction; Py: Person-years. HR: Hazard ratio. CI: confidence interval.

Cox models adjusted by all variables in the table, plus educational level and physical activity of moderate and high intensity, and stratified by sex and age.

^a^Defined as a self-reported diagnosis of diabetes or prescription of insulin or oral anti-diabetic medication.

^b^Normal weight: Body mass index (BMI) <24.9 kg/m^2^; overweight: 25 ≤ BMI < 29.9 kg/m^2^; obese: BMI ≥30 kg/m^2^.

^c^Heterogeneity by sex cannot be assessed because the lack of cases in the reference category for women prevents fitting a regression model.

Uncontrolled hypercholesterolemia and hypertension were prognostic of IHD but not stroke (albeit results were suggestive of a higher stroke risk among the hypertensive participants, this association remained non-significant), as shown in Table 3. As compared to healthy subjects, no increased CV risk was detected for previously diagnosed participants who showed cholesterol and blood pressure values below the clinical threshold.

**Table 3:** Risk of incident cardiovascular disease associated to hypercholesterolemia and hypertension in a cohort of adult population from Murcia (south-east Spain)

	Ischemic heart disease						Stroke		All events	
					
	Angina		AMI		All ischemic events					
					
	Py/cases	HR (95%CI)	Py/cases	HR 95%CI	Py/cases	HR 95%CI	Py/cases	HR 95%CI	Py/cases	HR 95%CI
**Hypercholesterolemia**^**a**^										
No	19194/12	1	19213/10	1	19170/19	1	19288/20	1	19113/37	1
Controlled	2609/5	1,22 (0,39-3,81)	2629/4	1,30 (0,39-4.37)	2606/7	1,18 (0,47-2,96)	2676/7	1,44 (0,56-3,67)	2578/11	1,08 (0,53-2,24)
Uncontrolled	3814/16	2,20 (0.94-5.16)	3813/22	3,57 (1,57-8.12)	3757/29	2,83 (1,49-5,37)	3913/9	1,08 (0,45-2,60)	3724/32	1.89 (1,11-3,23)
					*P*_*heterogneity by sex *_*= *0.789		*P*_*heterogneity by sex *_= 0.097		*P*_*heterogneity by sex *_= 0.217	

**Hypertension**^**b**^										
No	16631/7	1	16633/7	1	16614/12	1	16683/9	1	16580/18	1
Controlled	2043/4	3,32 (0,89-12,36)	2069/2	1,80 (0,36-9,17)	2040/5	2,71 (0,90-8,12)	2126/4	1,98 (0,56-6.98)	2037/7	2,22 (0,89-5,54)
Uncontrolled	7238/23	2,78 (1,10-7,08)	7248/28	4,06 (1,64-10.03)	7172/40	3,15 (1,55-6,40)	7341/24	2,31 (0.98-5,43)	7070/56	2,85 (1,60-5,06)
					*P*_*heterogneity by sex *_= 0.030		*P*_*heterogneity by sex *_= 0.995		*P*_*heterogneity by sex *_= 0.053	

AMI: Acute myocardial infarction; Py: Person-years. HR: Hazard ratio. CI: confidence interval.

^a^Controlled hypercholesterolemia defined as prior diagnosis of hypercholesterolemia or use of lipid-lowering medication and total serum cholesterol <240 mg/dl; uncontrolled hypercholesterolemia defined as total serum cholesterol ≥240 mg/dl.

^b^Controlled hypertension defined as prior diagnosis of hypertension or use of anti-hypertensive medication and systolic/diastolic blood pressure <140/90 mmHg; uncontrolled hypertension defined as systolic/diastolic blood pressure ≥140/90 mmHg.

The estimated population risk attributable to each CVRF (and 95%CI) is presented in Table 4. Smoking accounted for the largest proportion of IHD cases (45%), followed by hypertension (33%) and, to a lesser extent, hypercholesterolemia (22%). According to the formula PAR = 1 - (1 - PAR_risk factor 1_) × (1 - PAR_risk factor 2_) × ... × (1 - PAR_risk factor n_), it can be estimated that these three factors together account for 1 - (1 - 0.45) × (1 - 0.33) × (1 - 0.22) = 71% of IHD cases.

**Table 4:** Population risk of ischemic heart disease attributable to the major risk factors in a cohort of adult population from Murcia (south-east Spain)

Risk factor	**Prevalence of exposition among the population (%)**^**e**^	95% CI	**Relative risk**^**f**^	95% CI	Population attributable risk (%)	95% CI
Hypercholesterolemia^a^	27.5	25.3 - 29.7	2.02	1.13 - 3.62	21.9	2.1 - 41.7
Hypertriglyceridemia^b^	16.5	14.6 - 18.5	1.85	1.06 - 3.25	12.4	0 - 25.6
Hypertension^c^	22.2	20.3 - 24.1	3.21	1.61 - 6.42	32.9	10.7 - 55.1
Diabetes^d^	6.2	4.9 - 7.6	1.68	0.86 - 3.29	4.0	0 - 10.5
Ever smoker	58.7	56.1 - 61.4	2.41	1.18 - 4.91	45.0	14.9 - 75.1

PAR: Population Attributable Risk.

^a^Defined as total serum cholesterol ≥240 mg/dl, prior diagnosis of hypercholesterolemia or prescribed use of lipid-lowering medication.

^b^Defined as serum triglyceride concentration ≥150 mg/dl.

^c^Defined as systolic blood pressure ≥140 mmHg, diastolic blood pressure ≥90 mmHg, prior diagnosis of hypertension or use of anti-hypertensive medication.

^d^Defined as a self-reported diagnosis of diabetes or prescription of insulin or oral anti-diabetic medication.

^e^Prevalence in the population of Murcia (20-70 years) standardized to the 2001 census population of the region (data from the DINO Study, 2002).

^f^Relative risk estimated as the hazard ratio of Cox models adjusted by all variables in the table, plus educational level, body mass index, and physical activity of moderate or high intensity, and stratified by sex and age.

The results of the comparison of baseline characteristics between subjects finally included in the analyses and those who were lost to follow-up is presented as supplementary data [Additional File 1: Table S1]. Overall, there were no significant differences between groups except that lost to follow-up participants more frequently smoked and, despite no significant differences in serum cholesterol distributions were found, were less prone to be hypercholesterolemic than participants included in the analysis.

The age-standardised cumulative incidence rates of AMI and stroke in the Murcia population is shown in supplementary data [Additional File 1: Table S2]. Men suffered from increased incidence of both conditions to a larger extent than women. Generally men presented higher rates of AMI than stroke, whereas women showed low rates of AMI, and higher incidence of stroke. Truncated age-standardised incidence AMI rates per 100000 person-years were 403 in men and 124 in women for the 35-64 years age range. Incidence rates of stroke for the same age group were 243 and 221 per 100000 person-years for men and women, respectively.

## Discussion

CVD remain as the leading cause of mortality in Spain [20], and Murcia, despite its Mediterranean location, shows incidence and mortality rates of CVD above the Spanish average [2,21,22]. In the present study, age-standardised incidence AMI rates were higher than previously reported by the IBERICA Study (1997-1998), a population-based registry of AMI [2]. However, coronary mortality is decreasing in Spain, and the high rates found in the present study would partly reflect this more unfavourable CV pattern of the past decade [21,23].

Our analysis revealed that hypertension and tobacco use are the most important CVRF in Murcia. Furthermore, hypercholesterolemia and diabetes were associated to IHD, and hypertriglyceridemia to AMI. The baseline prevalence of hypertension in Murcia was below the country average, but there was a larger proportion of current smokers [3,24]. During the 1992-2002 period there was an overall favourable evolution of the CV risk profile of the population, with significant reductions in the prevalence of smoking and hypertension, increased percentage of people engaging in regular physical activity, and control of overweight prevalence in the region, in spite of which hypercholesterolemia increased by 30% [25]. However, changes in the exposition levels over time were not assessed in the members of the cohort and could not be accounted for in the analyses.

According to our results, the risk of IHD was two- to three-fold higher in subjects with high cholesterol or blood pressure values, ever smokers and diabetics. Of note, hypertriglyceridemia, a factor systematically excluded from CV risk charts and equations [8-10], was an independent determinant of AMI risk in this cohort of almost similar magnitude to that of serum cholesterol, in agreement with previous evidence in Spain [26]. On the other hand, information on diabetes was based on participants' self-reports, which are known to under-represent the true prevalence of the condition in our population [27]. Thus, the CV risk assessed for diabetes might be under-estimated and should be considered with caution. Virtually no data exist in Spain on the prospective association between CVRF and incidence of ischemic or cerebro-vascular events. A study by Marín *et al*. [13] found lower risk estimates than the present study, except for tobacco use (relative risk = 2.60, 95%CI: 1.75-3.85) in a sample of men and women attending a primary care centre. Lower relative risks were also reported by Tomàs i Abadal *et al*. [14] in a male occupational cohort from north-east Spain. However, large differences regarding the design, samples studied and follow-up periods preclude direct comparisons with the present study.

The major risk factors for stroke in this population were smoking and hypertension. Evidence for stroke associations with traditional risk factors exists in the literature [28], but as far as we know, this is the first prospective study in Spain to present risk estimations of cerebro-vascular disease by population levels of exposure to CVRF. Diabetes was not a significant predictor of stroke, although the possibility that such an association could be detected with a prolonged follow-up time and a larger number cases should not be discarded.

According to our results and most recent estimates of CVRF prevalence [5], 45% of IHD cases were attributable to smoking, 33% to arterial hypertension, and 22% to hypercholesterolemia (Table 4). Thus, hypertension displaces hypercholesterolemia as the second leading cause of CVD in this population. The limited number of IHD cases warrants caution in the interpretation and further generalization of results. Because of the high proportion of population in Murcia exposed to tobacco use, it would be expected from such initiatives as the toughening of legislation on smoking (which came into effect in the country since 2007) to have a large impact on cardiovascular prevention in the region as well as the rest of Spain. As compared with previous national estimates of PAR of IHD, it is remarkable the role of hypertension, as well as the lack of anthropometrical factors, among the population determinants of risk [7]. Multivariate adjustment by intermediate variables such as hypertension, hypertriglyceridemia and diabetes did attenuate the estimated HR for categories of BMI, although overweight and obesity neither were significantly associated with CV risk in a univariate model (data not shown). However, obesity was twice as high in stroke cases than in non-cases (50% *vs*. 21.8%; data not shown), suggesting that the detection of an excess risk associated with stroke in the obese might be constrained by limited statistical power. The lack of predictive ability of excess body weight on cardiovascular outcomes in the present study should not be an obstacle for considering obesity as one of the major health problems in the region, where its prevalence is among the highest in Spain and surrounding countries [29].

Some limitations should be considered. Despite the long follow-up time, the study lacked power to provide separate estimates for men and women, as it would be advisable in order to better account for sex differences in the morbi-mortality patterns of CVD [2,13]. The lack of information on potential confounding factors, such as diet or alcohol consumption [6,30], might also be a limitation. There were fewer hypercholesterolemics and more ever-smokers among participants lost to follow-up [Additional File 1: Table S1], but since we have no information on the cardiovascular outcomes in these participants we are unable to predict how this selection bias could have affected the results. One fifth (21.6%) of the initial sample refused to provide a blood specimen or to undergo a physical examination. Albeit few comparisons are possible due to lack of relevant biochemical and clinical information, these were in profile younger men, more educated, with lower BMI and more prone to engage in vigorous physical activity. No differences were observed in smoking patterns. Except for age (controlled for in the analyses), none of these factors was found to significantly predict CVD, and therefore such a selection bias would probably have had a minor effect on the results.

The study has several important strengths, as the population-based longitudinal design and the estimation of PAR using regional-specific risks and prevalence data, provides the first ever prospective results on the association between CV outcomes and CVRF in the Region of Murcia, and adds to the almost inexistent evidence yet available in Spain. Our results show the extent to which the effect of certain RF, particularly lipids, might be overlooked when analysed in regard to global CV endpoints, as in the calibrated SCORE charts[9] recommended for CV prevention in Spain [31]. We therefore support the development of specific CV risk charts for the primary prevention of IHD in Spain [8], and stress the need for their validation with regional longitudinal data that account for IHD variability in Spain.

In summary, our results provide prospective estimates of coronary, cerebro-vascular and total CV risk in Murcia, corroborate a relatively high incidence of AMI within the Spanish context and underpin the importance of promoting a non-smoking lifestyle as a key objective for CV prevention.

## Conclusions

Our study provides first-time population-based prospective estimates of IHD risk attributable to traditional CVRF in Spain. Smoking and hypertension accounted for most of the IHD risk, whereas hypercholesterolemia was third in place. However, despite its very high prevalence among the population, obesity was not a significant determinant of IHD or stroke in the region.

## Competing interests

The authors declare that they have no competing interests.

## Authors' contributions

JMH performed the statistical analyses and drafted the manuscript. MJT was involved in the study conception and design, coordinated the field work, participated in the analysis and drafting of the manuscript, and obtained all funding. DG was responsible for identification of new cardiovascular cases, provided critical revision of the manuscript and helped with the analysis and interpretation of data. CN was involved in the study design, critically revised the manuscript and made substantial contributions to its final content. All authors read and approved the final manuscript.

## Pre-publication history

The pre-publication history for this paper can be accessed here:

http://www.biomedcentral.com/1471-2458/10/640/prepub

## Supplementary Material

Additional file 1 **Tables S1 and S2**. Table S1. Comparison of baseline characteristics between the included participants and participants lost to follow-up. Table S2. Cumulative incidence rates of acute myocardial infarction and stroke per 100000 person-years in the adult population of Murcia (south-east Spain), by sex. Click here for file
