# Supplementary material for: Cardiovascular risk estimated after 13 years of follow-up in a low-incidence Mediterranean region with high-prevalence of cardiovascular risk factors
Source: BMC Public Health. 2010 Oct 25;10:640. doi: 10.1186/1471-2458-10-640 (PMC3091564; doi:10.1186/1471-2458-10-640)
Supplement: Additional file 1 — Tables S1 and S2. Table S1. Comparison of baseline characteristics between the included participants and participants lost to follow-up. Table S2. Cumulative incidence rates of acute myocardial infarction and stroke per 100000 person-years in the adult population of Murcia (south-east Spain), by sex. [file 1471-2458-10-640-S1.DOC]

**Additional File 1:**

## Supplemental Table S1 - Comparison of baseline characteristics between the included participants and participants lost to follow-up.

IQR: Interquartile range (Q_3_ - Q_1_).

^a^Controlled hypercholesterolemia defined as prior diagnosis of hypercholesterolemia or use of lipid-lowering medication and total serum cholesterol <240 mg/dl; uncontrolled hypercholesterolemia defined as total serum cholesterol ≥240 mg/dl.

^b^Controlled hypertension defined as prior diagnosis of hypertension or use of anti-hypertensive medication and systolic/diastolic blood pressure <140/90 mmHg; uncontrolled hypertension defined as systolic/diastolic blood pressure ≥140/90 mmHg.

^c^Defined as a self-reported diagnosis of diabetes or prescription of insulin or oral anti-diabetic medication.

^d^Participants with missing data on drug use, excluded from statistical tests.

## Supplemental Table S2 - Cumulative incidence rates of acute myocardial infarction and stroke per 100000 person-years in the adult population of Murcia (south-east Spain), by sex.

^a^Rates standardised to the population of the Region of Murcia according to the 2001 census.

^b^Rates standardised to the European Standard Population.

**Supplemental Table S1**

|  |  | **Participants included (N = 2023)** | |  | **Participants lost to follow-up (N = 291)** | | |
| --- | --- | --- | --- | --- | --- | --- | --- |
|  |  | **Median** | **IQR** |  | **Median** | **IQR** | ***P*** |
| **Age at recruitment (years)** | | 39.7 | 19.1 |  | 36.8 | 18.8 | 0.426 |
| **Body mass index (kg/m^2^)** |  | 26.5 | 5.6 |  | 26.1 | 6.0 | 0.084 |
| **Total serum cholesterol (mg/dl)** | | 190.0 | 64.0 |  | 184.0 | 56.0 | 0.127 |
| **Serum triglycerides (mg/dl)** | | 96.0 | 70.0 |  | 95.0 | 72.0 | 0.302 |
| **Systolic blood pressure (mmHg)** | | 125.0 | 24.0 |  | 121.0 | 27.0 | 0.051 |
| **Diastolic blood pressure (mmHg)** | | 79.0 | 15.0 |  | 77.0 | 14.0 | 0.206 |
|  |  |  |  |  |  |  |  |
|  |  | **N** | **%** |  | **N** | **%** | ***P*** |
| **Sex** | Men | 956 | 47.3 |  | 140 | 48.1 |  |
|  | Women | 1067 | 52.7 |  | 151 | 51.9 | 0.785 |
|  |  |  |  |  |  |  |  |
| **Hypercholesterolemia^a^** | No | 1456 | 72.0 |  | 228 | 78.4 |  |
|  | Controlled | 205 | 10.1 |  | 18 | 6.2 |  |
|  | Uncontrolled | 307 | 15.2 |  | 36 | 12.4 | 0.032 |
|  | Unknown^d^ | 55 | 2.7 |  | 9 | 3.1 |  |
|  |  |  |  |  |  |  |  |
| **Triglyceridemia** | <150 mg/dl | 1593 | 100.0 |  | 233 | 100.0 |  |
|  | ≥150 mg/dl | 430 | 27.0 |  | 58 | 24.9 | 0.605 |
|  |  |  |  |  |  |  |  |
| **Educational level** | Illiterate | 66 | 3.6 |  | 17 | 6.5 |  |
|  | Incomplete primary | 629 | 34.7 |  | 82 | 31.3 |  |
|  | Primary shool | 822 | 45.4 |  | 120 | 45.8 |  |
|  | Secondary school | 295 | 16.3 |  | 43 | 16.4 |  |
|  | University | 211 | 11.6 |  | 29 | 11.1 | 0.239 |
|  |  |  |  |  |  |  |  |
| **Hypertension^b^** | No | 1257 | 62.1 |  | 185 | 63.6 |  |
|  | Controlled | 161 | 8.0 |  | 25 | 8.6 |  |
|  | Uncontrolled | 572 | 28.3 |  | 77 | 26.5 | 0.776 |
|  | Unknown^d^ | 33 | 1.6 |  | 4 | 1.4 |  |
|  |  |  |  |  |  |  |  |
| **Diabetes^c^** | No | 1804 | 91.1 |  | 253 | 90.0 |  |
|  | Yes | 177 | 8.9 |  | 28 | 10.0 | 0.293 |
|  | Unknown^d^ | 42 | 2.1 |  | 10 | 3.6 |  |
|  |  |  |  |  |  |  |  |
| **Smoking** | Non-smoker | 1049 | 86.1 |  | 125 | 81.7 |  |
|  | Former smoker | 169 | 13.9 |  | 28 | 18.3 |  |
|  | Smoker | 805 | 66.1 |  | 138 | 90.2 | 0.018 |
|  |  |  |  |  |  |  |  |
| **Body mass index** | <25 kg/m^2^ | 706 | 45.0 |  | 120 | 52.2 |  |
|  | 25-29.9 kg/m^2^ | 863 | 55.0 |  | 110 | 47.8 |  |
|  | ≥30 kg/m^2^ | 454 | 28.9 |  | 61 | 26.5 | 0.103 |
|  |  |  |  |  |  |  |  |
| **Moderate physical activity** | None | 1744 | 92.0 |  | 249 | 89.9 |  |
|  | <1/2 h/day | 151 | 8.0 |  | 28 | 10.1 |  |
|  | ≥1/2 h/day | 128 | 6.8 |  | 14 | 5.1 | 0.287 |
|  |  |  |  |  |  |  |  |
| **Intense physical activity** | None | 1841 | 96.9 |  | 262 | 96.0 |  |
|  | <2 h/day | 59 | 3.1 |  | 11 | 4.0 |  |
|  | ≥2 h/day | 123 | 6.5 |  | 18 | 6.6 | 0.720 |
|  |  |  |  |  |  |  |  |

**Supplemental Table S2**

|  |  | **Acute myocardial infarction** | | |  | **Stroke** | | |
| --- | --- | --- | --- | --- | --- | --- | --- | --- |
|  |  | **Crude** | **Age-adjusted (95%CI)^a^** | **Age-adjusted (95%CI)^b^** |  | **Crude** | **Age-adjusted (95%CI)^a^** | **Age-adjusted (95%CI)^b^** |
|  |  |  |  |  |  |  |  |  |
| Men | 20-70 years | 239.6 | 230.1 (202.4 - 257.8) | 288.2 (254.2 - 322.1) |  | 179.4 | 251.5 (214.5 - 288.6) | 284.4 (244.5 - 324.3) |
|  | 35-64 years | 354.0 | 353.0 (310.2 - 395.8) | 403.4 (354.1 - 452.7) |  | 220.8 | 219.2 (185.3 - 253.1) | 242.5 (204.4 - 280.6) |
|  |  |  |  |  |  |  |  |  |
| Women | 20-70 years | 57.6 | 57.2 (44.6 - 69.9) | 75.6 (58.8 - 92.4) |  | 100.5 | 123.9 (102.8 - 145.0) | 151.5 (126.7 - 176.3) |
|  | 35-64 years | 97.0 | 106.9 (83.2 - 130.7) | 124.0 (96.4 - 151.6) |  | 156.8 | 195.8 (163.1 - 228.6) | 221.4 (184.3 - 258.6) |
|  |  |  |  |  |  |  |  |  |
| Total | 20-70 years | 142.4 | 144.1 (129.0 - 159.2) | 178.8 (160.3 - 197.3) |  | 137.4 | 173.8 (154.0 - 193.6) | 201.9 (180.2 - 223.6) |
|  | 35-64 years | 215.5 | 232.8 (207.9 - 257.6) | 262.6 (234.3 - 290.9) |  | 186.7 | 200.8 (177.9 - 223.6) | 227.0 (200.8 - 253.2) |
|  |  |  |  |  |  |  |  |  |
